# EARS2 significantly coexpresses with PALB2 in breast and pancreatic cancer

**DOI:** 10.1016/j.ctarc.2022.100595

**Published:** 2022-06-28

**Authors:** Steven Lehrer, Peter H. Rheinstein

**Affiliations:** a Department of Radiation Oncology, Icahn School of Medicine at Mount Sinai, New York, US; b Severn Health Solutions Severna Park, MD, US

**Keywords:** Oncogenes, Genetics, Pam50, Luminal b subtype

## Abstract

**Background:**

PALB2 (BRCA2 partner and localizer) is a BRCA2-interacting protein that is required for BRCA2 genome caretaker tasks and interacts with BRCA1. Women with PALB2 mutation have a 40% to 60% higher risk of breast cancer, almost equivalent to women who have BRCA mutations. PALB2 mutation may also increase the risk of pancreatic cancer. New guidelines for PALB2 mutation in breast cancer advise pancreatic cancer screening, which includes M.R.I.s of the pancreas as well as endoscopic ultrasonography, for women who have a family history of pancreatic cancer. Using the Cancer Genome Atlas (TCGA) and The Human Protein Atlas we examined genes that co-express with PALB2 in breast and pancreatic cancer.

**Methods:**

We used cBioPortal for Cancer Genomics to analyze data in TCGA. cBioPortal provides visualization, analysis and download of large-scale cancer genomics data sets. We used the UCSC Xena Browser to additionally analyze gene expression in TCGA.

**Results:**

Six genes, EARS2, ARL6IP1, DNAJA3, KNOP1, RPUSD1, and TMEM186, significantly coexpressed with PALB2 in both breast and pancreatic cancer. Glutamyl-tRNA synthetase 2 (EARS2) was the only gene coexpressing with PALB2 in the breast and pancreatic cancer subjects that was significantly related to pancreatic cancer survival. Elevated PALB2 and EARS2 gene expression are both significantly associated with the PAM50 Luminal B subtype and high risk of recurrence, suggesting why these women may need active intervention, such as prophylactic mastectomy.

**Conclusions:**

EARS2 expression might be a risk factor for pancreatic cancer in breast cancer patients with PALB2 mutations. By assessing EARS2 expression in breast tumors, the clinician might obtain a second piece of information that, with family history of pancreatic cancer, could inform the decision to perform pancreatic cancer screening.

## 1. Introduction

PALB2 (BRCA2 partner and localizer) is a BRCA2-interacting protein that is required for BRCA2 genome caretaker tasks and interacts with BRCA1. PALB2 causes Fanconi’s anemia by biallelic germline loss-of-function mutations (also known as FANCN); whereas monoallelic loss-of-function mutations are linked to an elevated risk of breast and pancreatic cancer. Women with PALB2 mutations have a 40% to 60% higher risk of breast cancer, almost equivalent to women who have BRCA mutations [1]. In addition, high expression of PALB2 predicts poor prognosis in patients with advanced breast cancer [2].

Unlike BRCA1 and BRCA2 mutations, which are frequently present in Ashkenazi Jews, PALB2 mutations are not seen in the Ashkenazi community. PALB2 has been linked to Finnish, French Canadian, and Greek women in some research [3].

Like BRCA1 and BRCA2 mutations [4], PALB2 mutation may increase the risk of pancreatic cancer [5]. For women who have a family history of pancreatic cancer, new guidelines for PALB2 mutation in breast cancer advise pancreatic cancer screening, which includes M.R.I.s of the pancreas as well as endoscopic ultrasonography. The new guidelines may improve treatment as well as prognosis [6], since PALB2 protein expression is an unfavorable marker in pancreatic cancer [7]. But the prevalence rate of PALB2 mutations in a non-BRCA1/2 breast cancer population specifically selected for a family history of pancreatic cancer did not appear to be significantly increased compared to that observed in other breast cancer populations [8].

Using the Cancer Genome Atlas (TCGA) and The Human Protein Atlas we examined genes that co-express with PALB2 in breast and pancreatic cancer. Evaluation of these genes might supplement information provided by family history of pancreatic cancer and inform the decision for pancreatic cancer screening.

## 2. Methods

TCGA is a project, begun in 2005, to catalog genetic mutations responsible for cancer, employing genome sequencing and bioinformatics. We used cBioPortal for Cancer Genomics to analyze data in TCGA. cBioPortal provides visualization, analysis and download of large-scale cancer genomics data sets [9]. Gene expression is quantitated as Fragments Per Kilobase of transcript per Million mapped reads upper quartile (fpkm-uq), which is an RNA-Seq-based expression normalization method [10].

We used the UCSC Xena Browser to analyze gene expression [11]. (https://xenabrowser.net). UCSC Xena allows exploration of functional genomic data sets for correlations between genomic or phenotypic variables.

We consulted The Human Protein Atlas to evaluate protein expression. The Human Protein Atlas is a Swedish-based project that began in 2003 with the goal of mapping all human proteins in cells, tissues, and organs by combining omics technologies, such as antibody-based imaging, mass spectrometry-based proteomics, transcriptomics, and systems biology [12]. The Human Protein Atlas allows examination of the expression of individual genes and how they affect patient survival in 17 different types of cancer. A computer-based modeling approach to cancer types in 8000 patients is provided. 900,000 patient survival profiles are available, including cancers of the colon, prostate, lung, pancreas, and breast [13].

Statistical methods: All tests of significance were taken from calculations by cBioportal, UCSC Xena, and The Human Protein Atlas. cBioPortal uses the Benjamini-Hochberg False Discovery Rate correction procedure for multiple comparisons [14], as in Table 2. Other statistical methods are described elsewhere [10, 12, 13, 15].

## 3. Results

Table 1 displays demographics and clinical details of subjects in the study. We evaluated 51 genes in the data sets that gave rise to EARS2 being the ONLY coexpressed gene in both breast and pancreatic cancers.

6 genes with expression most significantly correlated with PALB2 expression in breast and pancreatic cancer were EARS2, ARL6IP1, DNAJA3, KNOP1, RPUSD1, and TMEM186. Glutamyl-tRNA synthetase 2 (EARS2) was the only gene coexpressing with PALB2 in the breast and pancreatic cancer subjects that was significantly related to worse pancreatic cancer survival (Table 2).

**Fig. 1.**
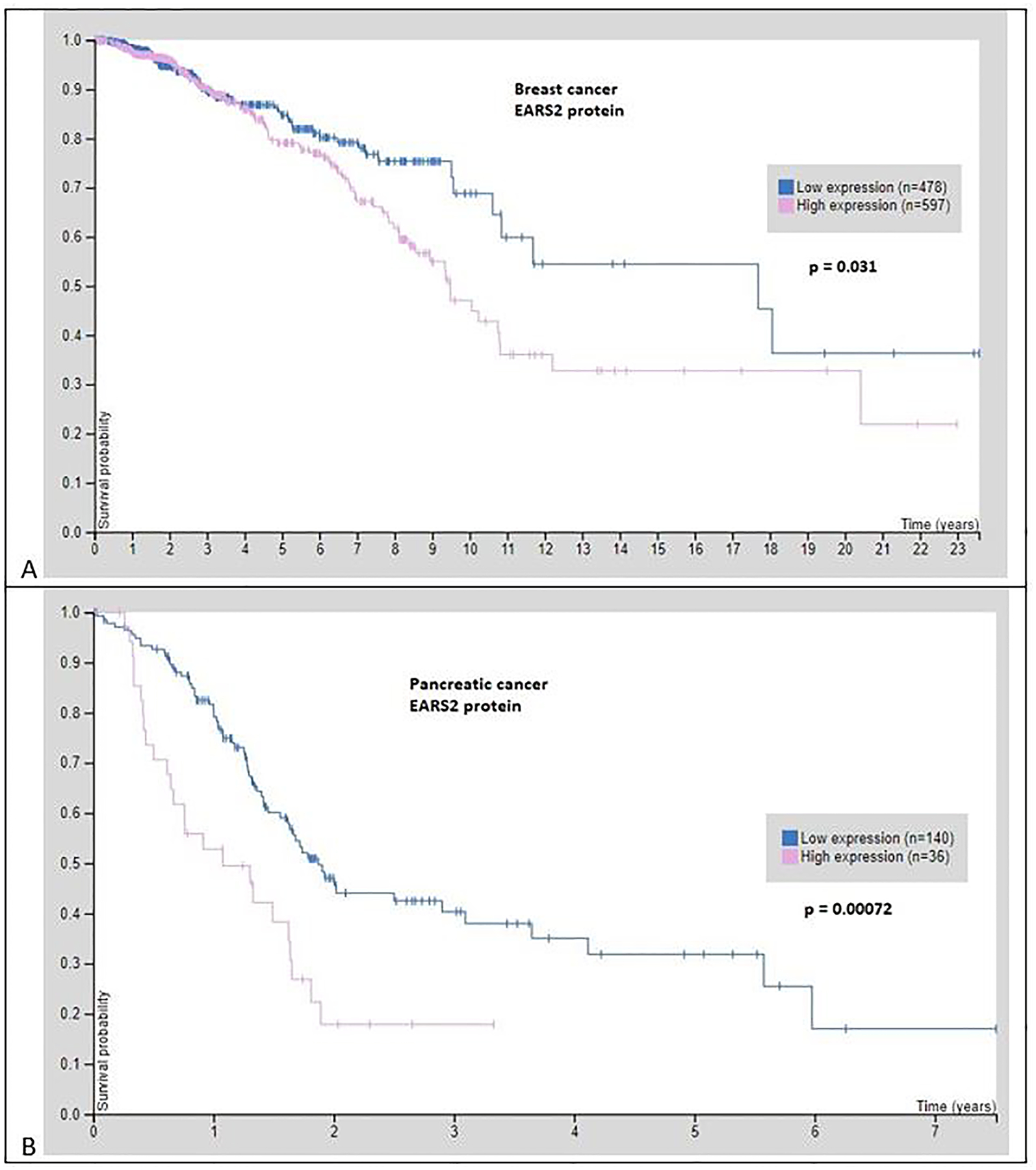
EARS2 protein expression and breast cancer survival. Higher levels of EARS2 protein correlated with worse survival (*p* = 0.031, log rank test). B. EARS2 protein expression and pancreatic cancer survival. Higher levels of EARS2 protein correlated with worse survival (*p* = 0.00072, log rank test). (The Human Protein Atlas). To separate low expression from high expression, samples are divided on the median.

**Fig. 2.**
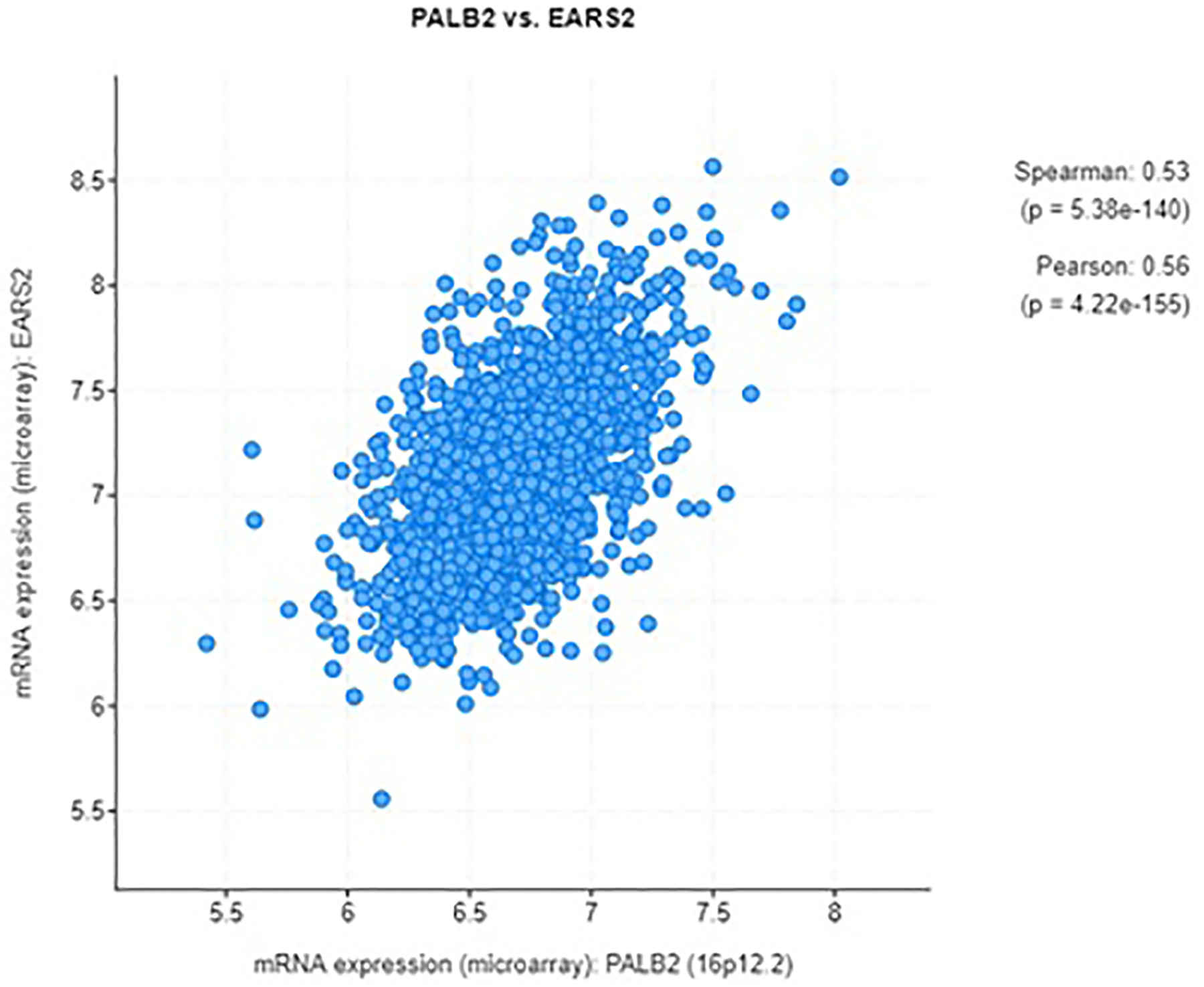
mRNA expression PALB2 versus EARS2 in 1904 cases of breast cancer. Expression of PALB2 and EARS2 was significantly correlated. (cBioPortal).

**Fig. 3.**
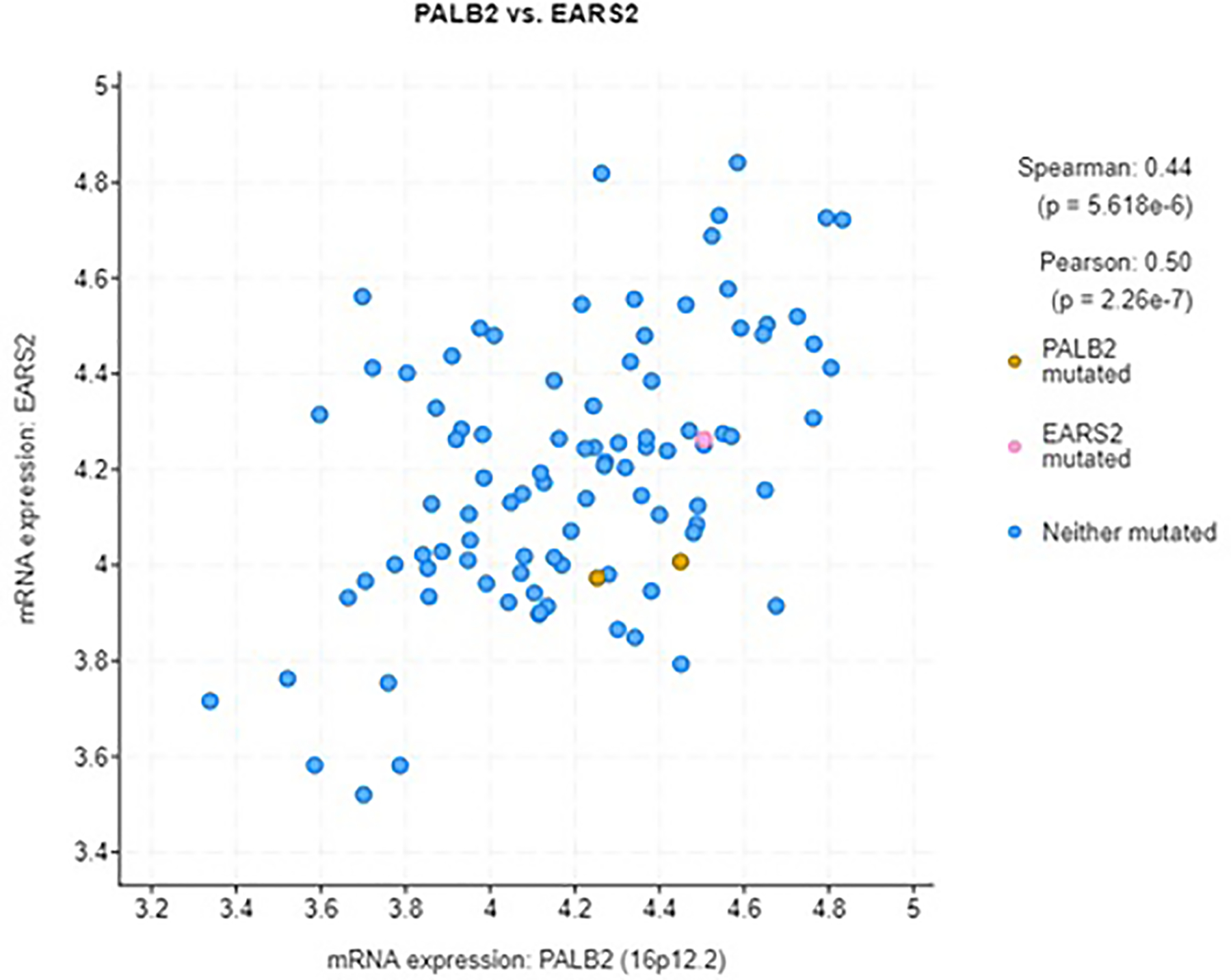
mRNA expression PALB2 versus EARS2 in 96 cases of pancreatic cancer. Expression of PALB2 and EARS2 was significantly correlated. In two cases PALB2 was mutated: one missense mutation of unknown significance and one truncating mutation, a putative driver. In two cases EARS2 was mutated: one inframe mutation of unknown significance (not shown) and one truncating mutation of unknown significance. (cBioPortal).

EARS2 protein expression affected breast cancer survival. Higher levels of EARS2 protein correlated with worse survival (*p* = 0.031, log rank test, Fig. 1A). Fig. 1B shows EARS2 protein expression and pancreatic cancer survival. Higher levels of EARS2 protein correlated with worse survival (*p* = 0.00072, log rank test). mRNA expression assessment of PALB2 versus EARS2 in 1904 cases of breast cancer revealed that PALB2 and EARS2 significantly coexpressed (Fig. 2) [16]. mRNA expression of PALB2 and EARS2 in 96 cases of pancreatic cancer was significantly correlated [17]. In two cases PALB2 was mutated: one missense mutation of unknown significance and one truncating mutation, a putative driver. In two cases EARS2 was mutated: one inframe mutation of unknown significance and one truncating mutation of unknown significance. The expression of EARS2 protein was dependent on PALB2 and vice-versa (Fig. 3).

**Fig. 4.**
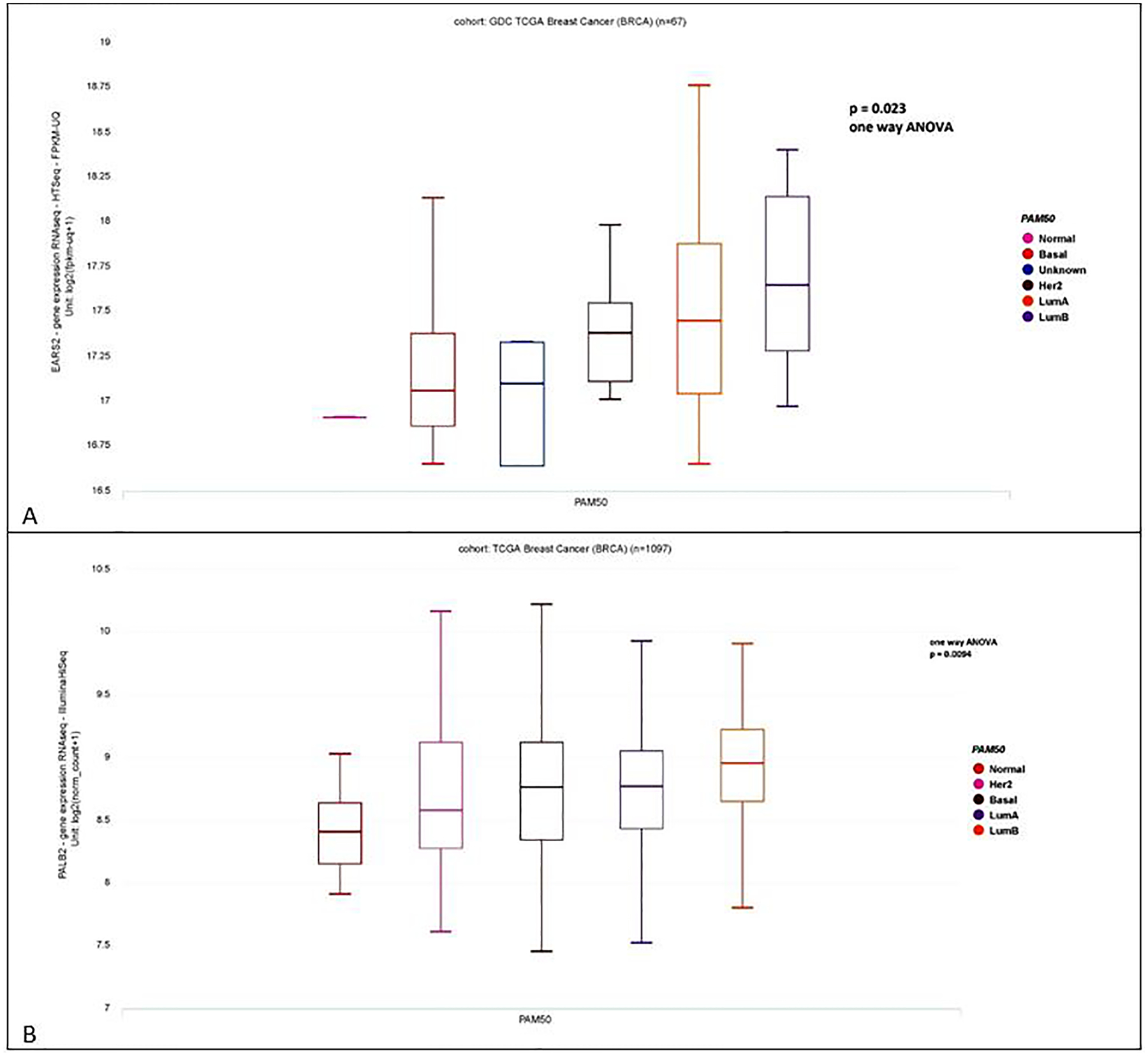
PAM50 versus EARS2 gene RNA expression, 67 breast cancer subjects, expression highest in Luminal B subjects (*p* = 0.023, one way ANOVA). (UCSC Xena) 4B PAM50 versus PALB2 gene RNA expression, 1097 breast cancer subjects, expression highest in Luminal B subjects (*p* = 0.0094, one way ANOVA).

PAM50 versus EARS2 gene RNA expression in 67 breast cancer subjects showed highest RNA expression in Luminal B subtype subjects (*p* = 0.023, one way ANOVA, Fig. 4A). PAM50 classification versus PALB2 gene RNA expression, 1097 breast cancer subjects, indicated that PALB2 expression was highest in Luminal B subtype subjects (*p* = 0.0094, one way ANOVA, Fig. 4B).

**Fig. 5.**
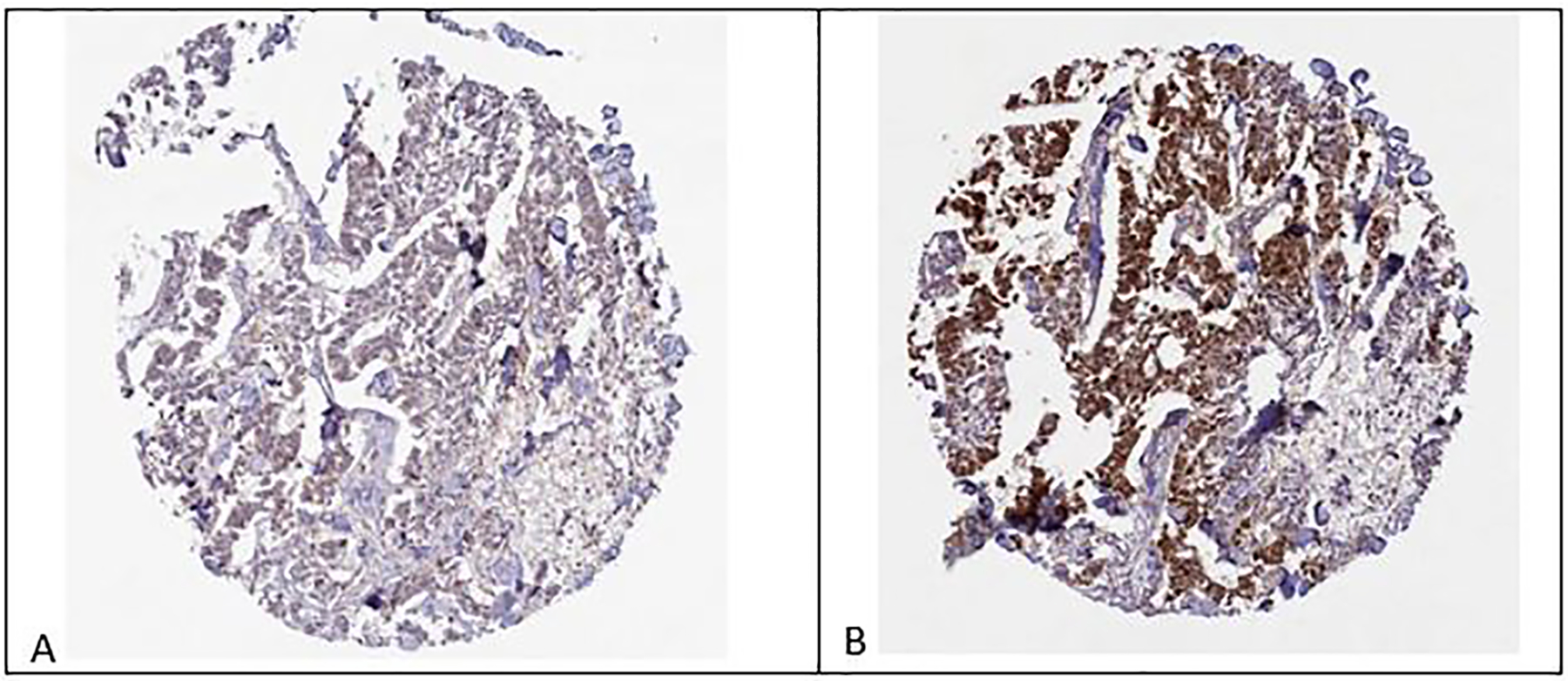
Female, age 83 Breast, ductal carcinoma, EARS2 staining with Antibody HPA043289 not detected. 5B Female, age 83, Breast, ductal carcinoma, EARS2 staining with Antibody HPA043633 high intensity. (The Human Protein Atlas).

Fig. 5A shows breast tissue, female, age 83, duct carcinoma, EARS2 staining absent. Fig. 5B shows breast tissue, female, age 83, duct carcinoma, EARS2 staining high intensity. Although these are discordant results for two different antibodies on what appears to be the same tissue core, the difference in epitopes (the part of an antigen molecule to which an antibody attaches itself) may be sufficient to explain the varied results.

## 4. Discussion

Six genes (BRCA1, BRCA2, CDKN2A, TP53, MLH1, ATM) have mutations that enhance a person’s risk of pancreatic cancer significantly. EARS2 is not among them. Because the six genetic alterations were found in patients with no family history of pancreatic cancer, genetic testing has been suggested as the new standard of care [18].

The EARS2 gene codes for mitochondrial glutamyl-tRNA synthetase. This enzyme is necessary for protein synthesis in mitochondria. Transfer RNA (tRNA) aids in the assembly of amino acids into a chain during protein synthesis in both the mitochondria and the cytoplasm. To the developing chain, each tRNA transports a unique amino acid. Mitochondrial glutamyl-tRNA synthetase binds glutamate to the appropriate tRNA, ensuring that glutamate is supplied to the correct location in the mitochondrial protein.

EARS2 mutations are found in individuals having leukoencephalopathy of thalamus and brainstem with high lactate (LTBL). At least 23 mutations in the EARS2 gene are responsible. LTBL subjects usually have issues with thinking and motor abilities, as well as ability to manage muscular activity. The EARS2 gene mutations that cause LTBL are thought to lower mitochondrial glutamyl-tRNA synthetase levels. A lack of this protein prevents mitochondria from properly assembling new proteins. Improper protein assembly causes mitochondrial energy production to be disrupted. The specific mechanism by which EARS2 gene mutations cause LTBL is unknown [19].

EARS2 is involved in Leigh Syndrome, a life-threatening neurological condition that usually manifests in the first year of life. This disorder is marked by a gradual loss of mental and motor abilities (psychomotor regression), and often leads to death within two to three years, usually from respiratory failure. A tiny percentage of people do not develop symptoms until they are adults, or their symptoms increase more slowly [20].

EARS2 is related to at least three forms of cancer. In renal cancer EARS2 protein expression is a favorable prognostic marker, whereas in pancreatic cancer EARS2 protein expression is an unfavorable prognostic marker [21]. In addition, EARS2 may be a predictor of colon and rectal cancer [22]. EARS2 expression in cancer can be assessed by tissue immunostaining, as in Fig. 5.

In most people, cancer gene mutations do not occur at random. Certain cancer gene mutations are frequently found together, suggesting that they may work in tandem to promote tumor growth and progression [9]. This may be the case with the co-occurring expression of PALB2 and EARS2. The relation of EARS2 protein expression with reduced breast cancer survival in Fig. 1A might be related to the increased risk of breast cancer in women with PALB2 mutations.

After accounting for tumor stage, grade, and age at diagnosis, PAM50 subtypes are linked with breast cancer outcomes after 15+ years of follow-up. The results are unaffected by menopausal status at the time of diagnosis. Women with the Luminal B subtype have a 60 percent higher risk of recurrence than women with the Luminal A subtype [23]. Elevated PALB2 and EARS2 gene expression are both significantly associated with the Luminal B subtype (Fig. 4), and suggest why these women may need active intervention, such as prophylactic mastectomy.

Our study has limitations. The reason to look for genes associated with both breast and pancreatic cancer in Table 2 is to focus on the most relevant genes. If a gene is strongly associated with pancreatic cancer but not breast cancer in Table 2 and is strongly associated with pancreatic cancer survival—not tested in these genes—it could be a potential risk factor for pancreatic cancer in breast cancer patients. In addition to examining the six genes identified in Table 2 as primary candidates, it would be worthwhile to look at the other genes as secondary candidates for pancreatic cancer risk factors. However, this could affect the q values and criteria for multiple tests of significance in Table 2.

Weaknesses in our study:

- There is the possibility of gain/loss of function mutations of PALB2 (rather than the mutations that would only affect expression levels) as we detected a very small number of mutations in our analysis.
- PALB2 mutations do not necessarily equate to significantly altered PALB2 expression. Our conclusion that EARS2 expression might be a risk factor for pancreatic cancer in breast cancer patients with PALB2 mutations is based on mRNA expression since there were only 2 pancreatic cancers and no breast cancers with a PALB2 mutation. But, as was mentioned above, high expression of PALB2 predicts poor prognosis in patients with advanced breast cancer [2].
- In pancreatic cancer PALB2 expression may be involved in pancreatic ductal adenocarcinoma cell migration independent of mutational status [24].
- Increased expression of homologous recombination (HR) genes such as PALB2 is associated with poor prognosis, high grade cancers, probably because HR genes are expressed predominantly in the S and G2/M parts of the cell cycle when HR occurs. Moreover, PALB2 expression correlates with markers of cellular proliferation and the E2F1 transcription factor (a marker for the S phase) [25]. Therefore, since EARS2 correlates with PALB2, EARS2 could also be a poor prognostic marker for high grade, more proliferative tumors, like Ki67, not directly related to poor outcome through EARS2’s mechanism of action.

In conclusion, EARS2 expression might be a risk factor for pancreatic cancer in breast cancer patients with PALB2 mutations. By assessing EARS2 expression in breast tumors, as in Fig. 5, the clinician may obtain a second piece of information that, with family history of pancreatic cancer, could inform the decision to perform pancreatic cancer screening. Further studies are warranted.

## Figures and Tables

**Table 1:** Demographics and clinical details of subjects in this study.

Breast Cancer		Pancreatic cancer	
N	1904	N	176
age	60.4 ± 13	age	65 ± 11
ER pos	73%	pancreatic adenocarcinoma	85%
ER neg	19.30%	male	61%
HER2 neg	69.10%	female	39%
HER2 pos	9.80%		
Histograde 1	9.60%		
Histograde 2	41.40%		
Histograde 3	44.50%		
Cellularity high	48.80%		
Cellularity moderate	37.90%		
Cellularity low	10.40%		
premenopause	20%		
postmenopause	80%		
hormone Rx	63.30%		
no hormone Rx	36.70%		
chemotherapy	82.20%		
no chemotherapy	17.80%		
invasive ductal	76.10%		
mixed ductal lobular	11.80%		
invasive lobular	7.90%		
invasive breast	1.80%		
zero nodes	51.60%		
1 node	16.60%		
2 nodes	8.30%		
3 nodes	5.50%		
4 nodes	2.50%		

**Table 2:** Genes with expression most significantly correlated with PALB2, leftmost 5 columns in breast cancer, rightmost five columns in pancreatic cancer. Six genes, EARS2, ARL6IP1, DNAJA3, KNOP1, RPUSD1, and TMEM186, had expression significantly correlated with PALB2 in both breast and pancreatic cancer. EARS2 was the only gene in the breast cancer and pancreatic cancer subjects that coexpressed with PALB2 and was significantly related to pancreatic cancer survival. Q value is derived from the Benjamini-Hochberg False Discovery Rate correction procedure for multiple comparisons. (cBioPortal).

Breast CaCorrelated Gene	Cytoband	Spearman’s Correlation	p-Value	q-Value	Pancreatic CaCorrelated Gene	Cytoband	Spearman’s Correlation	p-Value	q-Value
ARL6IP1	16p12.3	0.538	3.02E-143	5.45E-139	KNOP1	16p12.3	0.558	3.40E-09	5.65E-05
EARS2	16p12.2	0.533	5.38E-140	4.86E-136	ERI2	16p12.3	0.522	4.79E-08	3.99E-04
KNOP1	16p12.3	0.46	2.73E-100	1.64E-96	TMEM186	16p13.2	0.511	1.03E-07	5.73E-04
CEP20	16p13.11	0.451	6.17E-96	2.78E-92	ZNF646	16p11.2	0.495	2.91E-07	1.21E-03
LCMT1	16p12.1	0.433	1.22E-87	4.41E-84	HMOX2	16p13.3	0.477	9.19E-07	3.06E-03
DNAJA3	16p13.3	0.432	2.22E-87	6.66E-84	NMRAL1	16p13.3	0.465	1.85E-06	5.13E-03
KIF22	16p11.2	0.418	3.02E-81	7.78E-78	PARN	16p13.12	0.455	3.24E-06	7.69E-03
TUFM	16p11.2	0.417	6.17E-81	1.39E-77	CDC37	19p13.2	0.448	4.77E-06	9.91E-03
RMI2	16p13.13	0.417	8.03E-81	1.61E-77	EARS2	16p12.2	0.445	5.62E-06	0.0104
TRAP1	16p13.3	0.409	1.33E-77	2.39E-74	FAM32A	19p13.11	0.438	8.23E-06	0.0137
TMEM186	16p13.2	0.408	2.13E-77	3.49E-74	DNAJA3	16p13.3	0.436	9.05E-06	0.0137
CREBBP	16p13.3	0.401	1.67E-74	2.51E-71	CYSRT1	9q34.3	0.432	1.10E-05	0.0152
NUBP1	16p13.13	0.401	2.57E-74	3.56E-71	IQCK	16p12.3	0.429	1.31E-05	0.0166
POLR3K	16p13.3	0.395	3.36E-72	4.33E-69	OSBPL3	7p15.3	0.427	1.40E-05	0.0166
BFAR	16p13.12	0.39	3.02E-70	3.51E-67	ERCC4	16p13.12	0.422	1.82E-05	0.0202
UBFD1	16p12.2	0.39	3.12E-70	3.51E-67	TRAPPC10	21q22.3	0.421	1.96E-05	0.0203
COQ7	16p12.3	0.385	3.22E-68	3.42E-65	ARL6IP1	16p12.3	0.416	2.51E-05	0.0234
ZSCAN32	16p13.3	0.383	1.81E-67	1.82E-64	NMU	4q12	0.415	2.59E-05	0.0234
NFATC2IP	16p11.2	0.382	2.49E-67	2.36E-64	P2RX4	12q24.31	−0.415	2.67E-05	0.0234
REXO5	16p12.3	0.378	7.23E-66	6.52E-63	RRN3	16p13.11	0.411	3.21E-05	0.0267
TBL3	16p13.3	0.378	1.06E-65	9.11E-63	THUMPD1	16p12.3	0.408	3.70E-05	0.0282
UBE2I	16p13.3	0.378	1.28E-65	1.05E-62	RPUSD1	16p13.3	0.406	4.01E-05	0.0282
RPUSD1	16p13.3	0.377	1.96E-65	1.53E-62	TMEM154	4q31.3	0.406	4.04E-05	0.0282
SNRNP25	16p13.3	0.374	3.95E-64	2.97E-61	FAM86KP	4p16.1	0.406	4.06E-05	0.0282
FLYWCH2	16p13.3	0.373	9.50E-64	6.85E-06	GSPT1	16p13.13	0.404	4.37E-05	0.0291
